# Reflective, pragmatic, and reactive decision-making by maternity service providers during the SARS-CoV-2 pandemic health system shock: a qualitative, grounded theory analysis

**DOI:** 10.1186/s12884-023-05641-2

**Published:** 2023-05-20

**Authors:** Sergio A. Silverio, Kaat De Backer, Jeremy M. Brown, Abigail Easter, Nina Khazaezadeh, Daghni Rajasingam, Jane Sandall, Laura A. Magee

**Affiliations:** 1 Department of Women & Children’s Health, School of Life Course & Population Sciences, Faculty of Life Sciences & Medicine, King’s College London, 6th Floor Addison House, Great Maze Pond, Southwark, London, SE1 1UL UK; 2 Department of Women & Children’s Health, School of Life Course & Population Sciences, Faculty of Life Sciences & Medicine, King’s College London, 10th Floor North Wing, St. Thomas’ Hospital, Westminster Bridge Road, Lambeth, London, SE1 7EH UK; 3 Health Research Institute, Medical School, Faculty of Health, Social Care & Medicine, Edge Hill University, St. Helen’s Road, Ormskirk, L39 4QP Lancashire UK; 4 Chief Midwifery Office, NHS England and Improvement, Wellington House, 133-155 Waterloo Road, Southwark, London, SE1 8UG UK; 5 Maternity Services, St. Thomas’ Hospital, Guy’s and St. Thomas’s NHS Foundation Trust, Westminster Bridge Road, Lambeth, London, SE1 7EH UK

**Keywords:** COVID-19, Decision-making, Healthcare professionals, Health system shock, Maternity care, SARS-CoV-2 pandemic, Service reconfiguration

## Abstract

**Background:**

Pregnant and postpartum women were identified as having particular vulnerability to severe symptomatology of SARS-CoV-2 infection, so maternity services significantly reconfigured their care provision. We examined the experiences and perceptions of maternity care staff who provided care during the pandemic in South London, United Kingdom – a region of high ethnic diversity with varied levels of social complexity.

**Methods:**

We conducted a qualitative interview study, as part of a service evaluation between August and November 2020, using in-depth, semi-structured interviews with a range of staff (*N* = 29) working in maternity services. Data were analysed using Grounded Theory analysis appropriate to cross-disciplinary health research.

**Analysis & findings:**

Maternity healthcare professionals provided their views, experiences, and perceptions of delivering care during the pandemic. Analysis rendered three emergent themes regarding decision-making during reconfigured maternity service provision, organised into pathways: 1) ‘Reflective decision-making’; 2) ‘Pragmatic decision-making’; and 3) ‘Reactive decision-making’. Whilst pragmatic decision-making was found to disrupt care, reactive-decision-making was perceived to devalue the care offered and provided. Alternatively, reflective decision-making, despite the difficult working conditions of the pandemic, was seen to benefit services, with regards to care of high-quality, sustainability of staff, and innovation within the service.

**Conclusions:**

Decision-making within maternity care was found to take three forms – where at best changes to services could be innovative, at worst they could cause devaluation in care being delivered, and more often than not, these changes were disruptive. With regard to positive changes, healthcare providers identified staff empowerment, flexible working patterns (both for themselves and collectively as teams), personalised care delivery, and change-making in general, as key areas to capitalise on current and ongoing innovations borne out of the pandemic. Key learnings included a focus on care-related, meaningful listening and engagement of staff at all levels, in order to drive forward high-quality care and avoid care disruption and devaluation.

**Supplementary Information:**

The online version contains supplementary material available at 10.1186/s12884-023-05641-2.

## Background

The ‘COVID-19’ pandemic has posed an unprecedented threat to public health and human life. The outbreak spread rapidly across the world, with the UK reporting its first case on 31 January 2020 [1]. By 26 March 2020, the UK Government had legislated for national lockdown, with mandatory stay-at-home orders enforced across the four nations. These measures intended to reduce the spread of infection and associated hospital admissions [2].

To reduce the rate of infection, healthcare services globally reconfigured their healthcare provision, ensuring those most vulnerable had least exposure. Maternity services, in particular, were reconfigured to minimise the risk of SARS-CoV-2 to both pregnant women and their babies [3–9]. Challenges for maternity care-providers were numerous, including shortages of resource; these were physical (e.g., lack of beds as maternity wards were converted into ‘COVID-19 wards’) [10–12] and human (due to staff illness, self-isolation, shielding practices, or redeployment to provide other types of care) [3, 13, 14]. Further, there was rapid implementation of virtual care, in an environment which was not digitally-advanced [3, 13, 15–18].

In the UK, reconfiguration of maternity care services was documented in a nationwide audit which suggested a reduction in scheduled antenatal and postnatal care appointments, an increase in provision of virtual care, and at least temporary suspension of support for homebirths, continuity of care, and midwifery-led birthing units [14]. These changes were perceived as resulting in less personal, more fragmented care for women and their babies – antenatally, intrapartum, and postnatally [13, 19–26].

Throughout the pandemic in the UK, detailed guidance on continued delivery of safe and effective maternity care was provided by the Royal College of Obstetricians and Gynaecologists (RCOG) and Royal College of Midwives (RCM). Between January 2020 and January 2022, the RCOG and RCM issued 14 versions of their *‘Coronavirus (COVID-19) infection and pregnancy’*guidance [27]. Additional guidance on service reconfiguration and infection control in healthcare settings was issued by NHS England [28, 29] and the Institute of Health Visiting [30]. As such, a rapidly burgeoning mass of guidance was issued, to be absorbed, interpreted, and implemented by individual maternity care services and providers. Adding to the challenge was that these and other important guidance documents did not always agree or directly align – adding to the challenge of implementation.

To understand how those involved in providing maternity care experienced the pressures of rapidly reconfiguring their local care policies and provision during the SARS-CoV-2 pandemic, we interviewed a multi-disciplinary range of healthcare professionals as part of a service evaluation. This aimed to inform ongoing discussions regarding development of healthcare policy and guidelines aiming to build back a better maternity care service for future pandemic waves, post-pandemic recovery, and future health system shocks.

## Methods

### Details of ethical approval

This project was deemed a service evaluation by Guy’s and St. Thomas’ NHS Foundation Trust and was given Trust approval in July 2020 (service evaluation reference:- 11046).

### Study design

The overall study was designed as a service evaluation, the results of which have been published elsewhere [13]. As is often the case with qualitative data and with the nature of the pandemic circumstances, respondents provided much richer data than expected, allowing us to conduct subsequent analyses of the interview data. Therefore, whilst our study design was that of a service evaluation, the analytic technique presented in this paper is in response to the data quality being much higher than anticipated, which enabled a post hoc decision to be made to conduct the subsequent grounded theory analysis presented here.

The qualitative design chosen was semi-structured interviews with healthcare providers who had been involved in maternity services reconfiguration, either planning and/or provision. The full interview schedule can be found in Supplementary Material 1. Adopting a post-positivist research paradigm (i.e., the pursuit of objectivity in conjunction with recognition of the effect of socio-cultural pressures and biases) allowed us to focus on the emergence of theory which explained more than each individuals’ experience alone [31].

To this end, we present ourselves as ontologically critical realist (accepting of peoples’ lived realities as knowledge of the *‘truth’* even if it is not necessarily true) and epistemologically objectivist (a procedural attempt at the acquisition of new knowledge). Critical realist ontology [32] enabled an empathic reflexive judgement to reported experiences, with understanding of the structural conditions and social pressures as important context within which healthcare providers were working. We adopted an objectivist epistemological stance [32], with the interviewers and analysts positioned as objective outsiders to the system within which respondents worked, as neither the interviewer [SAS – experienced qualitative researcher, KDB – junior qualitative researcher] nor analysts [SAS, KDB, JMB] were employees of the Trust. The philosophical underpinning lent itself best to a grounded theory analysis, which is rooted in ontological critical realism and epistemological objectivism.

### Respondent recruitment

A critical case purposeful sampling technique [33] was used to identify 29 healthcare providers working in maternity services. As appropriate for this service evaluation study design, we recruited from one NHS Trust (hospital), with the aim of extrapolating findings to other contexts [33].

To reflect the breadth and balance of professionals providing maternity care, we sent out directorate-wide e-mails to maternity care staff. Expressions of interest in taking part in interviews were directed to a non-clinical member of the service evaluation team [SAS], to preserve the anonymity of potential respondents from clinical leadership within the Trust. All respondents confirmed their willingness to participate at the beginning of each interview recording. Respondents were made aware their identity would remain anonymous, but that their de-identified data would be shared with the Trust and prepared for publication and other such dissemination. Respondents’ transcripts were anonymised during the transcription process and assigned a number according to the order in which they were interviewed. Full characteristics of respondents are detailed in Table 1*.*

**Table 1:** Description of respondents

**Characteristic**	**Respondents** ***N*** **= 29 (%)**
*Professional Background*	
Midwifery	12 (41·4)
Obstetrics	6 (20·7)
Health Visiting ^a^	3 (10·3)
Other Medical Specialisms (e.g. Internal Medicine)	2 (6·9)
Anaesthesia	2 (6·9)
Neonatology	1 (3·4)
Nursing	1 (3·4)
Imaging Sciences	1 (3·4)
Clerical	1 (3·4)
Maintenance/Cleaning/Security ^b^	0 (0·0)
*Position (Level of Seniority and/or Primary Responsibility)*	
Frontline Clinician (e.g. Junior Doctors, Midwives)	14 (48·3)
Senior Clinician (e.g. Consultants)	4 (13·8)
Clinical Manager (e.g. Clinical Staff responsible for delivery of a team)	4 (13·8)
Strategic Leadership (e.g. Clinical Staff with Senior Management Responsibilities)	4 (13·8)
Research (e.g. Clinically trained staff whose main role is to deliver clinical research)	2 (6·9)
Administrative (e.g. medical secretaries and office managers)	1 (3·4)
Maintenance/Cleaning/Security ^b^ (e.g. service staff)	0 (0·0)
*Years of Experience (Mean* = *16·2 years)*	
> 5 years	3 (10·3)
6–10 years	7 (24·1)
11–20 years	10 (34·5)
21 + years	9 (31·0)
*Years of Experience at this Trust (Mean* = *9·4 years)*	
> 5 years	10 (34·5)
6–10 years	10 (34·5)
11–20 years	9 (31·0)
21 + years	0 (0·0)
*Redeployed *^*c*^	
Yes	7 (24·1)
No	22 (75·9)
*Age (Mean* = *44·6 years)*	
18–24	0 (0·0)
25–34	5 (17·2)
35–44	10 (34·5)
45–54	10 (34·5)
55–64	4 (13·8)
≥ 65	0 (0·0)
*Sex*	
Female	26 (89·7)
Male	3 (10·3)
*Ethnicity *^*d*^	
White (White British, White Irish, White Gypsy/Traveller, White Other)	19 (65·5)
Black (Black African, Black Caribbean, Black Other)	5 (17·2)
Asian (Bangladeshi, Chinese, Indian, Pakistani, Asian Other)	3 (10·3)
Mixed (Mixed White/Asian, Mixed White/Black African, Mixed White/Black Caribbean, Mixed Other)	2 (6·9)
Other (Arab, Any Other)	0 (0·0)
*Has had a COVID-19 Positive Diagnosis *^*e*^	
Yes	8 (27·6)
No	18 (62·1)
Possibly (Unconfirmed)	3 (10·3)
*Clinically Vulnerable to COVID-19*	
Yes	2 (6·9)
No	27 (93·1)
*Clinically Vulnerable Household or Immediate Family Member*	
Yes	4 (13·8)
No	25 (86·2)

^a^In the UK, Health visitors are nurses or midwives who have undertaken additional training in community public health nursing to become specialist community public health practitioners

^b^Whilst recruitment was also open to members of staff from maintenance, cleaning, and security, we were unable to recruit any respondents from these aspects of the service

^c^Respondents were only deemed to have been redeployed when they had been asked to work in a clinical area where they had not previously worked as part of their contracted role at the Trust, or where their rotational working pattern had been completely re-designed due to COVID-19 service delivery reconfigurations

^d^ Ethnicity was defined by respondents in response to the question: *“Could you tell me the ethnicity with which you identify?”* and then grouped according to UK Government population statistics categories

^e^Respondents were recorded as ‘Possibly (Unconfirmed)’ when they believed they had contracted COVID-19, but never received clinical diagnosis

### Data collection

Between August and November 2021, two authors [KDB, SAS] conducted interviews (*N* = 29) via Zoom video-conferencing software [34], due to UK Government-imposed lockdown and physical distancing restrictions related to the COVID-19 pandemic. Data collection and analysis followed procedures for best practice for qualitative researchers in the field [35]. Interviews lasted 28–79 min (*Mean*= 50 min), were recorded, and the audio was professionally transcribed. Interviews were semi-structured, ensuring certain questions were asked of all respondents to allow for comparable analysis across the dataset, but still flexibile enough to allow interviewers to follow-up on points raised which were unique to each individual respondents’ experience [36]. Interviews followed a chronological order, covering respondents’ experiences of service reconfiguration over the course of the COVID-19 pandemic.

### Data analysis

We followed an approach to grounded theory methodology appropriate for cross-disciplinary health research [37–40]. This approach involves three analytic phases: coding, theme development, and theory generation [37], undertaken continuously, so data were analysed as soon as interviews were transcribed, rather than waiting until all interviews had been completed. Grounded theory analysis has long been used in studies of health, illness, and healthcare provision, but often is subject to disciplinary siloes [37]. Given the cross-disciplinary nature of our team, which included expertise in psychology [SAS, AE], social science [SAS, JS], midwifery [KDB, JS, NK], medicine [LAM, DR], and clinical education [JMB, DR], the approach set out by Silverio and colleagues [37] in 2019 has been widely accepted in the field as a remedy to the difficulties a team might face when using grounded theory for cross-disciplinary analyses.

Interviews were conducted until theoretical saturation was reached [41], a point identified through constant comparison of each new transcript coded with previously-analysed transcripts. Whilst theoretical saturation was reached with 18 respondents, anomalies remained in the data provided by respondents who were not from midwifery or obstetric backgrounds, and we were aware of the lack of ethnic diversity in the dataset. Following grounded theory, theoretical sampling can be undertaken when respondents with a particular characteristic exhibit experiences different from the main [38]; this facilitates determination of whether dataset anomalies are related to a particular group, or simply specific to an individual respondent [42]. Therefore, we theoretically sampled on these two characteristics (i.e., different professions and ethnicity). Full theoretical saturation was achieved with 29 respondents.

All coding of transcripts was conducted ‘by hand’ using Microsoft Word, which enabled multiple analysts to access the same transcript, remotely. Each transcript was first open-coded, line-by-line [KDB], using pertinent parts of the respondents’ own speech to provide a code for each line or sentence. Focused coding followed [SAS, KDB], where line-by-line codes were grouped into higher order codes representing trends in the data. These focused codes were merged, split, and rearranged to develop ‘super-categories’ [37]. At this point, a third analyst [JMB] re-coded approximately 15% of the transcripts as a reliability check, using just the super-categories; this analyst was otherwise masked to the original lower and higher order coding. Reliability of super-categories was confirmed between analysts. Finally, themes were developed by collapsing and re-arranging super-categories, and where required, offering themes with more appropriate names.

Following grounded theory methodology [37], the theory was subjected to within-team defence, twice, when it was further refined, ratified, and approved unanimously; this ensured that no other explanations were evident. Presented here are analyses addressing the individual experiences of providing maternity care during the pandemic; analyses addressing the system-level response to reconfiguring services during the pandemic has been published elsewhere [13].

## Analysis & Findings

Our analysis comprises three emergent themes about those providing and organising maternity care, and all centred on decision-making—the deliberate step-by-step process of gathering information, making choices, and assessing alternative resolutions: 1) ‘Reflective decision-making’, which resulted in unique opportunities for service improvement and satisfaction as care-providers; 2) ‘Pragmatic decision-making’, which was recognised as justifiable to at least some degree, but which resulted in disruption to care; and 3) ‘Reactive decision-making’, which resulted in devaluation of care. Data are presented in narrative prose, with the most illustrative quotations selected. Further supporting quotations are presented in Table 2. The final theory is then described, before being interpreted in relation to existing literature.

### Reflective decision-making → Unique opportunities for service improvement

Respondents discussed decision-making which was reflective, and involved interpretation of issued policy for their maternity population, and how maternity services should be reconfigured:

*“It was pretty cool to see on the whole the way the hospital adapted and responded. You could see that a lot of planning, a lot of thought had gone into it, and it was quite cool to see. I mean, obviously there’s always going to be areas it could do better, but I mean how often do you have a global pandemic? Hopefully not very often.”* (Midwifery Frontline Clinician)

Many respondents agreed on how this time for reflection facilitated innovation:

*“Zoom and MS Teams has revolutionised the way we do meetings within the NHS.”* (Anaesthetic Frontline Clinician)

*“The one thing we have to never lose again in the NHS was before Covid the patients always came first, which is right, but the staff never came second. The wellbeing part of caring for our staff really came to the forefront during Covid. Suddenly we had wellbeing zones, we had tea bags, we had coffee, we had spaces to go and relax. Covid needed to happen to bring those things to our wards and services.”* (Midwifery Clinical Manager)

And often this innovation was regarded as being free of ‘small-p’ political influence:

*“…it’s just really scrutinising services and deciding what’s necessary and what’s not. Something that was probably necessary and overdue but unable to be done before because of political limitations, which were somehow freed when you have a pandemic and a crisis. So that was positive.”* (Obstetric Frontline Clinician)

*“I think one of the key logistical changes has been that for the first time ever, we’ve been allowed - even encouraged - to work from home, which is quite an odd thing to do…”* (Neonatology Senior Clinician)

Many discussed how this type of decision-making, allowed for continued delivery of efficient and effective, high-quality care – across the service from antenatal sonography to postnatal health visiting:

*“We have been able to have longer appointment times, so you haven’t got the stress of fitting everything into a short space, then having the next patient come in. Not having visitors, we feel a bit less pressure. We can do our jobs and have good communication, but without the showmanship that we have had to do before.”* (Imaging Services Frontline Clinician)

*“…one thing I would say is this Covid has brought the best out of most services. Things that we didn’t really know that we could do before…”* (Health Visiting Clinical Manager)

Respondents often reported change was overdue, usually positive, and therefore welcome:

*“…the best thing, the best thing about this whole lockdown thing is joining up care stuff. So, clinic’s done this great thing where women can have a walk-in whooping cough [vaccination] now when they’re coming for their appointment. So, they’re coming in for their scan, they can have their bloods done, blood pressure done, have the whooping cough, go home. We’re trying to join up if they’re seeing Diabetes and they have to see the Optician, it’s all done at the same time. If they’re having a scan that morning, they see the Obstetrician that day and go home, so it’s not like two, three, four appointments in one week. If we can keep that up, that would be amazing because I think that is very helpful for the women.”* (Midwifery Frontline Clinician)

This also meant respondents often felt a sense of individual growth as they felt less pressured in their role:

*“That was hugely professionally and personally satisfying. Bizarrely, it was a good experience for me that allowed me to grow significantly both professionally and personally…”* (Obstetric Strategic Leader)

This was echoed in examples of how the service was seen to collectively grow in collaboration and collegiality:

*“In the first surge there was a pandemic, let’s all step up to the mark, this is proper medicine. We were all prepared to do what we needed to do so there was an awful lot of, ‘Tell me what you need me to do.’ Even if they wanted me to do ITU, I would have done it.”* (Anaesthetic Frontline Clinician)

*“…when we changed the way that we were working, so when some midwives were shielding, we moved the teams, working in different teams and I really enjoyed that actually. I really enjoyed meeting up with different colleagues, because in community we can be quite isolated actually. We generally don’t have the opportunity to go to big meetings, say the updates on the service or whatever, we generally don’t have the time to do that. So yes, to be working with different colleagues was great. Yes, I really enjoyed that.”* (Midwifery Frontline Clinician)

### Pragmatic decision-making → Disruption of care

Nevertheless, pragmatic decision-making led to some disruption in care which was perceived negatively, but was tolerated. On occasion, these decisions were perceived as focussed only on safety, in the narrowest sense of prevention of mortality from COVID-19 infection (which had been the priority of the RCOG and the RCM guidance, as opposed to the NHS England guidance). This meant staff often struggled to believe the care they were providing was sufficient:

*“I think the reduction in touch and closeness I think is actually quite important for both staff and the patients because there is healing in touch I think and also not being able to see faces because you are wearing a mask all the time now, I think is quite difficult both for patients and for clinicians. So, it reduces the amount of empathy you can convey. And also, it’s again like email and text, easy to misconstrue what is being said because you can’t see the whole face, because we are not only obscured by masks, but we are also obscured by eye wear as well. So yes. I think that’s probably the most unfortunate thing for me…”* (Obstetric Senior Clinician)

*“…they can rely on you to advocate for them and it’s just so much harder when there’s so much stuff in the way, you just look like you are going in to fumigate something and you already looked weird in so much plastic and goggles and things and then you can’t even do your normal care that you would.”* (Midwifery Frontline Clinician)

It was emphasised that the introduction of virtual care was within a digitally ill-equipped NHS unable to deliver video-care across the service, meaning that much virtual care was provided by telephone:

*“…not everyone is virtually savvy or there is some digital inequality or digital divide that often hasn’t been thought through……… for some mothers actually the virtual clinic may seem quite intrusive because where they live may not… They may not necessarily open it up to the clinical staff and suddenly they are exposed to that and I think it could make some of them uncomfortable.”* (Obstetric Senior Clinician)

*“The initial thing was we weren’t set up for it at all. I.T., things like headsets, cameras, which we still don’t have, and the administrative support for it. It was a huge amount of work that we didn’t have the admin support or structures for.”* (Obstetric Frontline Clinician)

The decision to disband continuity of care completely, rather than considering how it might have been delivered virtually, was ill-received:

*“…the loss of continuity of care I think is the thing that I have missed the most because you can be much more reassuring if you are the midwife that the woman sees all the way through, you can obviously address all of her issues, she gets to know you, you get to know her, there’s a feeling of......... connection, I think. So that has been lost.”* (Midwifery Frontline Clinician)

*“We need to scrap the idea that one size fits all and if this is what comes from the pandemic it’s that you can have some people who like virtual appointments, some people who prefer a face-to-face antenatal class; some people like coming to groups, some people prefer to have one-to-one breastfeeding support. That’s okay; we can create a service that meets all of those needs. We just have to be allowed to do it. We have to target the women in the right way so that we actually serve a purpose, rather than doing something because we have always done it.”* (Midwifery Clinical Manager)

### Reactive decision-making → Devaluation of care

There was a strong sense that the care provided was not good enough and that staff and service-users required reassurance that the current levels of care provision was the best approach, even when those providing the reassurance were unsure themselves:

*“…a lot of my time was about providing reassurance and trying to guide people when I didn’t feel I had the right guidance myself, because as you know, the guidance kept changing consistently. So, it was a bit of the blind leading the blind really, in the sense of reassuring staff…”* (Midwifery Strategic Leader)

Many clinicians often reported feeling like a ‘mouthpiece’ for the policy and guidance, assuring patients this was the correct care they should be offering, even when they did not believe it aligned with their views of what constitutes quality of care. Furthermore, the challenge of the service-provider fatigue quickly set-in:

*“There was lots of group forming and storming, which developed from the logistical nightmare of all of us trying to use different platforms and accessing shared files. Shared files this and shared files that… It became quite stressful. Personally, I felt a huge amount of guilt because I could see what was happening to my colleagues at work, but I didn’t have any more hours to work.”* (Midwifery Clinical Manager)

*“When people got tired and the initial exuberance wears off, people got quite stressed, cross, and upset with each other…”* (Obstetric Frontline Clinician)

There was a strong sense amongst staff who took part in the study, of the heavy burden of being the last stop for women’s care in their maternity journey, which only weighed heavier when the feeling was that quality of care had suffered:

*“I feel quite strongly that we didn’t get it quite right. The reason we have antenatal care – postnatal as well, all of it, all the care we normally do – is because women and babies are at risk in pregnancy and the early postnatal period. That is why we exist as midwives. Our job is to safeguard the women from developing problems. We spot things and get them attended to appropriately in a timely manner. The change in the schedule of care such that there was such a tiny amount of face-to-face appointments – certainly when we were deep in Covid – didn’t account for the balance of risk to women and babies…”* (Midwifery Frontline Clinician)

Often, staff discussed the challenges of delivering a service to comply with new regulations for safety. This was challenging given the pace and extent of changes to government, NHS, and Royal Colleges’ guidance, which were often received and interpreted as edicts by many:

*“Over a thirteen-week period, we had three-hundred different bits of guidance which were either optional or mandatory from NHS-E. We had to process them!”* (Obstetric Strategic Leader)

Many respondents focused on the immediate ‘climb-out’ of the pandemic circumstances.

*“I would want the service not to overload us, not to expect us to go back right now to some kind of normal way of working because we need more time. We do need more time.”* (Midwifery Frontline Clinician)

This often came hand-in-hand with respondents considering whether or not they could continue in their clinical roles in the wake of the pandemic, to deliver a para-pandemic service, before we entered a post-pandemic era:

*“It’s made me feel uninspired… Bored with the sort of… It’s like walking through treacle. Yes. I love, I love this job. I love it. I think it’s amazing. What a service! I feel so passionate about it……… but we can’t progress, or we can’t do any of it. It’s just uninspiring because everything takes so long and I just think, God… we’ve run a marathon, we are on to our second or third marathon now, we haven’t had our hot bath and cocoa yet. I feel like leaving, but there’s no way to go………. I think it’s difficult the last couple of weeks, we are getting a sense… the whole of the country is getting a sense of 'Oh God, here we go', and it’s starting to get polarised and difficult to just, you know, calm, and hold your own. I can’t hold my own forever…”* (Health Visiting Clinical Manager)

Others reflected on what these reactive decisions would mean for the future of maternity care delivery, as ultimately it was those clinicians at the coalface of providing care who bore the heavy burden of concern when they believed care was sub-optimal, and bore the brunt of concerns, challenges, and complaints when care was perceived poorly by patients themselves:

*“…we had an instability within the Midwifery leadership and that didn’t help, which showed how fragile our systems are and how dependent we are on the people rather than the system. So, that certainly shows that we are technically ill-prepared, because although we have fantastic individuals, services run on the individuals rather than on the operational system, and so therefore they’re not robust, and quite unsafe really.”* (Midwifery Strategic Leader)

**Table 2:** Supplementary Quotations

**Reflective decision-making → Unique opportunities for service improvement**	**Pragmatic decision-making → Disruption of care**	**Reactive decision-making → Devaluation of care**
*“That is really bad that it took a pandemic to get the wellbeing staff, but now we have a psychologist that works alongside our staff and we have psychological support for the staff. If they have an adverse incident then there is a lot more support for them. We have to never lose that again. We have to always keep the wellbeing of staff at the forefront…”* (Midwifery Clinical Manager) *“Everyone handles their burdens differently, but on the whole I think people felt like they were able to go in and share and say, “How are things for you?” There was a really good support network colleague to colleague.”* (Midwifery Frontline Clinician) *“I felt that there was good support. I never felt alone and I never felt that there wasn’t anyone I could have asked and it felt like our department really came together…”* (Research Midwife) *“And I think it was actually good to go into hospital where you see people. In a way, working from home would have been, probably, more isolating. At least there was some kind of normality. But yes, there was definitely a group of people there who you did stick together and… Yes, very supportive so you felt very supported with your colleagues in the department, I would say…”* (Nursing Frontline Clinician) *“There’s lots of positive things, but turning things round and changing the way we do things has been quite tiring. It is like anything. Some people cope well with change, some people are very resistant and haven’t enjoyed it. Trying to cope with all your various staff reactions to everything is difficult.”* (Obstetric Frontline Clinician) *“I think in that sense it worked really well, and I felt much more part of a team than I had before, really.”* (Research Midwife) *“At the beginning I was really up for it. I felt, especially as a part-time worker, that I was doing my duty and I was helping out…”* (Midwifery Clinical Manager) *“We bumbled together. Despite the fact it was a very traumatic clinical time, it was one of the best times of my leadership career.”* (Midwifery Strategic Leader) *“I think actually for a long time what we did was focus on patients, patients, patients, patients and we completely forgot the needs of the staff and we weren’t paying attention to the fact that happy staff means that you are more likely to get happy patients and now we’ve reaped the consequences of leaving staff out of the picture and I think, I hope, that COVID has flipped that, and we now understand. And I think that was one of the good things is the level of wellbeing provision provided to the staff, but it wasn’t enough to fully mitigate the concerns that have been raised by staff since COVID, but it was never going to because we did too little too late.”* (Obstetric Senior Clinician) *“…we met every single week and we had real comradeship about, 'Look, this is what we are doing, and this is how we are doing it”. We really supported each other.'* (Internal Medicine Senior Clinician) *“I think that one of the things that we’ve learnt from this pandemic is that maintaining distance within healthcare environments has been really challenging, and the reason for that is that our healthcare environments are far too crowded”* (Neonatology Senior Clinician) *“I always have multiple projects going on and I don’t like hanging about, so for me it was actually exciting that we could decide, and we could implement it, so that was refreshing in that sense for me.”* (Midwifery Strategic Leader)	*“PPE creates a barrier because you are wearing a mask and a lot of communication is through that face to face. That made it very difficult, and I think that created barriers really to communicating and connecting with women. It was the trust really I think it affected; I would say.”* (Midwifery Frontline Clinician) *“We were trying to focus on so many different things and not necessarily getting any of them right. Now we maybe get a couple of things right and not many other things…”* (Midwifery Clinical Manager) *“ There’s a reason we do it the whole way through and the care that we give normally is the bare minimum of what we think is safe, so for us to cut back on that, like that’s why we do what we do, we try to run an efficient healthcare system, so knowing that you are then not seeing those women as often, you know that the care that you are giving is not the ideal… it’s not optimum care. So, there was definitely a real worry, one that things would get missed.”* (Midwifery Frontline Clinician) *“I think it was the way it was brought into us, being told that it was just going to happen straightaway, I think there was a little bit of a feeling of oh, well, is it not a worry for us being overheated and hot anymore?”* (Midwifery Frontline Clinician) *“…it felt that there was a real possibility that I would feel in a position where I wasn’t able to give what I felt was the kind of care I would want to because I’m not used to being in that area and there could be very little support because we are in the middle of this huge pandemic.”* (Research Midwife) *“Women are going through probably one of the most profound experiences of their life when they generally have some feelings of vulnerability however happy to be pregnant they are and all of a sudden a) they were living in this global pandemic and no one really knew what that meant for people who were pregnant, but also it meant that all of the things that it would normally be absolutely a given that you would share with someone and have support with, suddenly people were being asked to do on their own…”* (Research Midwife) *“…we have been quite innovative. First of all what needs to stay is the fact that things have been a lot quicker to get approved. If someone has a guideline it gets approved overnight whereas there was a lot of tape initially. That has been a positive: we know we can get things approved a lot faster. There has been a bit more initiative in adopting to become more virtual. We have set up education classes for our caesarean section pathway online now. Things like that…”* (Obstetric Frontline Clinician) *“It would just be that change in virtual, but we know how to do it because we did it last time. Going forward we have now got the skills. We know everyone needs a headset. We realised and we have started doing patient education online so if there is a second wave, one group has already done it so the other groups can copy and keep going. We will probably get a little more savvy if there is a second wave because we will be working to keep patients at home when possible. We will get more into patient virtual education a bit better. We didn’t have time last time.”* (Obstetric Frontline Clinician) *“It comes back down to seeing someone face to face, having a midwife appointment and being able to go, ‘I have this question that I want to ask you,’ which they might not ask if they weren’t seeing someone face to face or it was on the telephone. It is that human contact bit that would be important.”* (Obstetric Frontline Clinician) *“So, women are often seeing someone different every time, there’s no trust being built up, a lot of questions I can only imagine are going unanswered and you’d have thought, 'oh great', with online maybe there are going to be longer appointments. But is that happening? No, of course not. Then as soon as there’s internet issues or connection issues it’s automatically putting up more barriers between women and them feeling okay to ask questions and things.”* (Midwifery Frontline Clinician) *“I think it’s made me feel I’m more adaptable to change than I thought, having to adapt to all these different working systems and *etc*. I’d like to be able to give clinical care virtually again, I’ve enjoyed that, having more contact with the women.”* (Research Midwife) *“…what women are missing out on is tapping into a midwife’s brain. That corridor conversation when you are in a clinic of, ‘Oh yes, I was going to ask you…’”* (Midwifery Clinical Manager) *“I think from women’s perspective, I can imagine that it is very disjointed, because you don’t get to experience coming into the hospital, you don’t get to experience that with your partner.”* (Midwifery Clinical Manager) *“I think for me in a specialty or in a clinical situation where you need to be looking at body language and tone of voice and facial expression and the non-verbal cues, doing it by phone or non-verbal I think we are giving a substandard service….. I think that since we, to some degree, do need to deliver a virtual offering, it ought to be a good virtual offering, so face to face virtually rather than simply auditory contact and that means that our I.T. needs to be upgraded – that old chestnut again – to provide that level of service.”* (Obstetric Senior Clinician) *“…our other problem is that our GPs have, to some extent, gone AWOL and have taken themselves out of the picture so that women can’t get their drugs and they are coming to the hospital to do that when actually things like ongoing prescriptions should be provided within the community”* (Obstetric Senior Clinician) *“The biggest thing I am unhappy about – it is ongoing – is the change in the schedule of care and the lack of face-to-face contact.”* (Midwifery Frontline Clinician) *“I see the psychological impact on women is really traumatised, really upset, because it is more than the physical. It is not just the physical checking the baby is okay, and the placenta is in the right position. It is a really big emotional bonding moment, the start of a family if it is their first baby…”* (Midwifery Frontline Clinician) *“If we are cutting down the relationships and the time with women and we are not building relationships with us, they don’t know us, they don’t know who they can turn to, that risk is going up – the very real, big risk. COVID is a risk too.”* (Midwifery Frontline Clinician) *“I think it’s a fundamental human right for fathers to be there, particularly to see their babies, and for someone to make a blanket decision that you simply cannot come in, I think there will be a backlash. So those kind of approaches we need to think through and sometimes it’s a matter of saying what can we do which is safe and right rather than what is the easy thing for us to do.”* (Obstetric Senior Clinician) *“…actually, the virtual clinic, really I worry that it may not be as effective because you really need a very judicious administrative closure of the loop and I don’t think that happens very well, so it’s very difficult from that aspect…”* (Obstetric Senior Clinician) *“I think the clinical aspect, there should still be more face to face and interactive, particularly for maternity where you need to palpate the baby. I think this has been, to some extent, imposed on mothers without really understanding how they feel about it and I’m sure if you ask most mothers or even a mother with a baby, actually the interactions make a difference to them and it just seems so isolating for your team to be contacting you by phone or virtually.”* (Obstetric Senior Clinician) *“I feel it was a compromised offer [of care], but we did the best in the circumstance. But it wasn’t our optimal.”* (Health Visiting Strategic Leader) *“…virtual [care] is good. But virtual [care] is fine if you able to have the means and resources to do that, but for a large proportion of our population English is the second language, there’s digital poverty.”* (Health Visiting Strategic Leader) *“…the idea of false compliance, because we were unable to go into their house to do home assessments and see whether there is maybe a health and safety issue or whatever thing that we need to discuss with them, or even if for example, those ones who are going through DV, maybe the perpetrator is still in the house or some other things. But before when we go in, we have to do that. But on the video, you don’t know.”* (Health Visiting Clinical Manager) *“…there’s certain camaraderie that happens when you just think… you know, you have to sort of surrender to it, but the thing is it’s that fine line between surrendering and just thinking, oh well, we can only do what we can do and then suddenly you are in complacency and there’s an incident and you think, oh my god, I took my eye off the ball for a minute there…”* (Health Visiting Clinical Manager) *“The FFP thing… we were quite lucky because we didn’t have to wear full FFP3, which was a real godsend. I got given one of the really big masks and you can’t understand what people are saying through them. It is all about communication. It is about eye contact, the real subtle things. That has been a detriment. Family photos. We take nice photos when the women have their babies. People wearing face masks – that has had an effect. Most patients understand and it is not a big deal, but there are subtleties lost with the PPE.”* (Anaesthetic Frontline Clinician)	*“I guess it would be really helpful if the messages were always the same, I think that’s the hardest thing. You’d hear something from the Government one day, then we’d hear something new at work, and it felt like we were guided by what the Government was saying. I just find that really frustrating at times, because I feel like we’ve got some real experts here that are worried about people’s health, not financial stuff. And if you have a universal message.”* (Midwifery Frontline Clinician) *“I’ve dropped my hours again because I don’t feel that the service is keeping me safe.”* (Midwifery Frontline Clinician) *“I can’t imagine anyone ever saying that they thought it would be okay that people wouldn’t be able to have someone with them throughout their labour and in the postnatal period and that was kind of suddenly just accepted that that was okay and I’m not sure still now that that is okay because actually one person who is well staying with that person, would that have really increased the risk of virus transmission hugely and would not having that impact massively on people’s long-term mental health?”* (Research Midwife) *“…the morale of a lot of people will change if we can’t ensure that the work/life balance is better.”* (Obstetric Frontline Clinician) *“I think we functioned similarly to pre-COVID, it was just a different staffing, a different work pattern. We held up all the services we provide. Nothing stopped. It was just the way it was delivered was a little different.”* (Obstetric Frontline Clinician) *“I was expecting at least at work to be my bubble and that we would be able to get on better looking out for each other more, but unfortunately I found the opposite and quite a stark difference to how it was before. As I mentioned, working in the office sometimes I found difficult because whether it is personality or the dynamics that were going on, that became much more obvious during the pandemic. It really has destroyed the team dynamics almost completely…”* (Imaging Sciences Frontline Clinician) *“It has been stressful trying to keep up with all the changes that were taking place in the trust or nationally and the concern that I wasn’t being protected with PPE as well as I could have been. At the beginning there were much stronger protective measures that were put in place, then as time went on it became less and less, but the virus is still the same, so you wonder: how effective is this that I am doing? I have always felt like I have had to do more than what was put out in the trust.”* (Imaging Sciences Frontline Clinician) *“It works much better if they are in clinic face to face and you can do LanguageLine because it makes you slightly worry about: are you missing anything?”* (Obstetric Frontline Clinician) *“I felt a responsibility to give them [friends] correct information, but I also didn’t really have a lot of information and it kept changing, so I just felt frustrated by that.”* (Midwifery Frontline Clinician) *“…so much of being a midwife and looking after a woman is about that rapport and being able to see each other’s faces and communication and non-verbal communication and so much of it is taken away when you have that physical barrier in front of your mouth, so it’s much harder to communicate and to get across empathy. So I think from the woman’s point of view, there must be a lot of stuff that is lost in translation and there’s a lot of empathy and compassion giving that’s probably not received because it can’t be given as well as it would usually.”* (Midwifery Frontline Clinician) *“…a lot of my colleagues were quite visibly so, so on already a fast-paced, high-risk environment where labouring women can go from 0 to 100 in two seconds, it just added more [of the same], I suppose, and colleagues were, I guess, a bit more pre-occupied with other things as well and life and home and the news.”* (Midwifery Frontline Clinician) *“…the first six weeks were extremely stressful, trying to manage the new reality. And then the way I split it in my head is the first six weeks were very stressful, the next six weeks were very boring [laughs]. Because once the stress had passed the variety of the clinics disappeared. Not seeing people face-to-face was boring.”* (Internal Medicine Frontline Clinician) *“Some women have preferred the fact that they don’t have to come in. I think for those women who don’t look after themselves as well as they should it has sometimes been an excuse for not looking after themselves even more, whereas perhaps we would have brought them in on a weekly basis…”* (Internal Medicine Frontline Clinician) *“…we coped with for a while because we all felt like we were doing the right thing. And then it got tiring and then we started to get burnt out. I started to get burnt out. I tapped into the psychological support, the e-mails that we got were lovely, and then it started to wear thin. And it still is wearing thin.”* (Midwifery Clinical Manager) *“For women who are more vulnerable, who would shy away, who don’t want to face it alone, then it’s probably not a very good service. And then they just turn up and have a baby. It’s lonely, ultimately. I think it’s a lonely service.”* (Midwifery Clinical Manager) *“…they can’t see your face; you can’t see their face and in women who have drug and alcohol situations or mental health situations that’s unsatisfactory. I also have lots of women with eating disorders. If you don’t see them, you can’t make a true assessment. Often, you’d call them, and they wouldn’t answer and they wouldn’t call back if they didn’t answer and you left a message, so it’s pointless, therefore the follow-up processes were also sub-optimal.”* (Obstetric Senior Clinician) *“…we’ve given less good care because we haven’t seen the women, we’ve missed things like growth restrictions… with the opportunity to miss things like growth restriction, pre-eclampsia sometimes and things like that.”* (Obstetric Senior Clinician) *“[on telephone clinics] It is less time-consuming. That is the only advantage I can think of, but I don’t really see it as an advantage.”* (Midwifery Frontline Clinician) *“We are not putting women at the centre of our care in this. Maybe that is the right decision. I am just a little midwife, I am not a person who is looking at the bigger picture, but I came into midwifery because I am a feminist and I believe in supporting women. In my training I learned that you put a woman at the centre of your care and everything else goes around that. I don’t see that is what is happening, but maybe in the bigger picture we are because we are trying to safeguard the whole population and women are part of that. I do see that there is more than one side of this.”* (Midwifery Frontline Clinician) *“I think that clearly there’s lack of acceptance that there may be a digital divide, so for some women I think it may actually cause harm and we’ll know later…”* (Obstetric Senior Clinician) *“…and I think that dictum that keep COVID patients away from the NHS to save the NHS, certainly in pregnancy, didn’t help!”* (Obstetric Senior Clinician) *“I found it mentally and physically exhausting to balance everything from a distance whilst shielding. And even in the early days. Yes. I was running on empty at times…”* (Health Visiting Strategic Leader) *“We didn’t have enough PPE at that time, and for me and my staff, the way I see it, maybe during that time when I was running around to make sure that they were safe and they had enough PPE, maybe that was when I contracted this virus myself.”* (Health Visiting Clinical Manager) *“…it’s been a challenge. I mean, for me, personally, I think I really struggled with trusting that the team are doing what they should be doing whilst they are not in the office, but then there’s no reason to suspect, and I think that’s often related to when I’m feeling a bit out of control, like I haven’t got tabs on things and I don’t know who is doing what and then I will have a call from a nurse who is in tears because she’s like, 'I can’t sit in my bed and do a case conference about horrendous things and then just log off and I’m sitting on my bed that I sleep in, it’s not working'. So that aspect of not having a boundary has been really tough emotionally for them.”* (Health Visiting Clinical Manager) *“[on banning partner visiting] It was a little draconian and a bit cruel on women.”* (Anaesthetic Frontline Clinician) *“There were a lot of women’s lives that were put at risk because we were having to do probably unnecessary PPE and resuscitation guidelines. Women’s lives were put at risk from limitations as opposed to my life being put at risk.”* (Anaesthetic Frontline Clinician) *“[on mixed messages for pregnant women] There are only so many times a woman would hear a maternity message in the day or a hospital message saying, ‘Please come to hospital if you are unwell,’ as opposed to the number of times they would hear a disaster message, death and destruction and everything, the NHS can’t cope – that ‘Leave the NHS alone’ message. They were fighting a losing battle ultimately.”* (Anaesthetic Frontline Clinician) *“It is the apathy and the frustration. We have done it before, and we didn’t like doing it before.”* (Anaesthetic Frontline Clinician) *“For the proportion of women that needed those assessments, probably the standard of their care went down because they weren’t being seen face to face for the Anaesthetic antenatal assessments. It is harder to pick up on cues about what exactly a woman is worried about, where exactly a woman might be reassured by things you might say to her.”* (Anaesthetic Frontline Clinician) *“The things I haven’t enjoyed about it, the things I found quite personally challenging, have been in the midst of it the relentlessness of it. […] Alongside that, the lack of any tangible holiday or break has been very hard because everything was cancelled in Covid. There was no annual leave, there didn’t seem any point in taking annual leave because you couldn’t travel. […]Even if you did, that all-pervasive Covid headlines are hugely draining in any healthcare professional because you are wondering what is coming next and when it is all going to change again, when is the sand going to shift, when are you going to be asked to do more or change again? 'How can I prepare, how can I get everything ready?' There is this baseline of tension.”* (Anaesthetic Frontline Clinician) *“…you realise is staff have got incredibly complex social circumstances themselves, and therefore that resilience within your workforce is not there at the same levels, so if something like this happens then I think that’s when you realise how fragile your workforce is.”* (Midwifery Strategic Leader) *“The balance between we have got to get on with it, to this is the best way of doing it, to this is just good enough, is really hard for some of our clinicians. Our clinicians are used to – generally, by and large – being able to provide really high-quality care. That was a real hit to them, but not something we could control…”* (Obstetric Strategic Leader) *“What I found the most difficult thing to navigate was the conflicting advice from… Say for instance the Institute of Health Visiting versus the NHS England guidance versus the PHE [Public Health England] guidance versus the [local] guidance, so trying to assimilate all the various guidance and protocols and service delivery mechanisms, trying to make sense of it and then distilling it into a standard operating procedure for each service where they could understand what was expected of them, when, how and where, doing what……… It was the all-consuming part of my role at that time”* (Health Visiting Strategic Leader)

## Discussion

### Summary

Our study centres on the professionals providing maternity services in one South London hospital, throughout the early stages of the SARS-CoV-2 pandemic (January-November 2020). We heard positives and negatives, both those of which individuals were accepting as necessary given the circumstances, and those that were not. However, our healthcare providers identified clear and irrefutable opportunities for positive change, ranging from staff empowerment, flexible ways of working individually and in teams, personalised care delivery, and change-making in general. It is time to capitalise on these learnings, so that staff providing care do not feel burdened by providing care they believe to be sub-optimal, are motivated by innovation, and avoid feeling like they are in a ‘parrotocratic’ situation whereby they are simply repeating policy handed down to them by senior Trust and Governmental sources, for whom they are expected to be an obedient mouthpiece.

### Main findings

The emergent theme of reflective decision-making allowed for staff cohesion, a sense of community, and opportunities for service improvement and innovation which were underpinned by principles of delivering high-quality and safe care. This echoes other research [43], especially from other high-income countries, which has demonstrated that when staff were able to voice concerns about services during the pandemic, they are more likely to work cohesively to deliver a service in which they had confidence [44–46], and in which the common goals are shared amongst management and frontline staff [47].

Next, staff understood the necessity of pragmatic decision-making, even when they acknowledged the potentially negative impact. Our findings have a variable impact of virtual care on patient experience is in-line with other research, suggesting virtual care was enjoyed by some [48]. Most staff commented how the service was not ready to be challenged by such a significant shock, and unprepared, such as with regards to digital technology. Others who endorsed these concerns regarding the inconsistent application of care provision, explained there could be adverse psycho-social, emotional, and physical health consequences for women and for their healthcare providers [49, 50].

Finally, the theme of reactive decision-making was exclusively supported by data which perceived this way of making decisions as negative. At best, staff reported only being able to provide the basic-level of care, but more concerning, was the reported devaluation of care which staff often suggested led to sub-optimal and even unsafe levels of care for women, their families, and their babies. This mirrors work carried out through the pandemic [51, 52] especially where pregnant women [53], new mothers [9, 20], and those who experienced a pregnancy loss or whose babies had died [26], have reported their care as not meeting their expectations or being of poor quality [21, 54, 55].

**Fig. 1:**
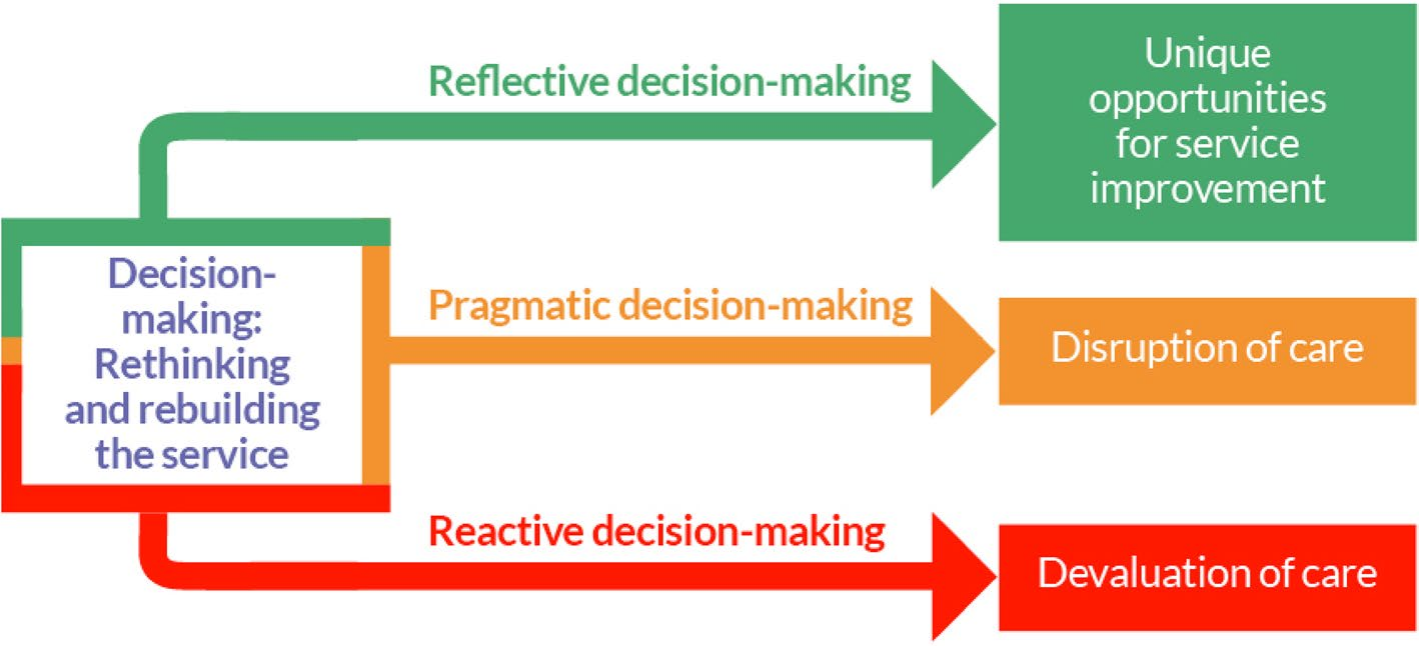
Final theory

Through Grounded Theory analysis of these data, a theory emerged about decision-making and care reconfiguration during the pandemic: ‘Decision-Making: Rethinking and Rebuilding the Service’ (Fig. 1).

This theory enables us to distinctly hypothesise about the workings of healthcare professional staff with regard to their decision-making processes, in response to a health system shock. Our theory suggests that in the face of adversity where the pandemic health system shock brought about the cessation of the ordinary provision of care, maternity healthcare professionals in this Trust acted to rethink and rebuild the service and care they had once provided. From our analysis, these processes of rethinking and rebuilding were undertaken with three distinct ways of decision-making: reflective, pragmatic, and reactive – with varying consequences, effects, and outcomes on the service provision. The theory helps to explain how maternity care services might have been altered during the pandemic, and lends insight into how maternity care and other healthcare services may weather future health system shocks, by capitalising on innovations which emanate from reflective decision-making, embracing pragmatic decision-making with minimal disruption to care, and – where possible – avoiding rethinking and rebuilding services using reactive decision-making.

### Interpretation

The SARS-CoV-2 pandemic has not been an easy health system shock to navigate for anyone in maternity care – policy makers, healthcare professionals, or women. However, our analysis has rendered clear outcomes with regard to future policy and practice. Firstly, when issuing guidance and its updates, consideration is needed of the balance required of the need for up-to-date information, with both the need for clear, consistent messaging (particularly when time is short) and the time required to implement change. Following a more reflective process should help to sustain high-quality care, and improve staff morale throughout health system shocks [56], such as the SARS-CoV-2 pandemic.

Furthermore, it is clear from our results that staff wish to be engaged in care policy and planning as well as delivery, including in the process of rapid change which must be implemented at pace (i.e., re-development, re-organisation, and re-deployment) – which echoes other research [57]. Whilst clinical staff are willing to accept pragmatism to some degree, at the heart of their work and their motivation for remaining in the role, is the provision of high-quality care, and many felt an immense burden to do so, even under the toughest and most uncertain of working (and global) circumstances. The health system relies on the frontline staff to provide a dynamic assessment of day-to-day work, and senior administrative staff taking decisions in the absence of understanding the clinical landscape, are no substitute for the input of those at the coalface.

Finally, maternity care requires consistent provision [9, 58], so services should not be reconfigured to significantly reduce human resources for provision of maternity care, particularly deployment of maternity staff away from delivery of maternal healthcare, especially in circumstances where staff are likely to have to have time off work, when sick and in need of isolation, shielding, or recovery [59]. Health impact assessments can be used to minimise the negative impacts of reconfigurations, particularly on those most vulnerable in the population, or those who already find services difficult to access [60–65].

### Strengths, limitations, & future research

This work was designed and undertaken as part of a portfolio of work in rapid response to the SARS-CoV-2 pandemic, and as such a major strength lies in capturing those early perspectives of the pandemic reported in real-time as the service reconfigurations were taking place. Full strengths and limitations of this portfolio have been documented elsewhere [13, 57], however, we highlight how we recruited a wide range of professional backgrounds, reflective of maternity service provision, with a mix of seniority, years of experience and years spent at the Trust, as well as a range of ethnicities; we suggest this goes some way towards countering the limitation which could be raised concerning recruitment only from one hospital. We acknowledge having a disproportionate number of women represented in this evaluation, although this is broadly reflective of maternity healthcare professionals, within and outwith the UK.

## Conclusion

Western culture has frequently interpreted the Chinese term for ‘crisis’ as being composed of characters for ‘danger’ and ‘opportunity’, though the latter is more correctly transliterated as: ‘change point’. The SARS-CoV-2 pandemic has posed a clear and irrefutable danger, both direct from the virus and indirectly from some of the social and healthcare service reconfigurations which were made in response. The pandemic had provided opportunities for innovation, disruption, and devaluation of care. These differing opportunities which arose from rethinking and rebuilding the healthcare service and care provision was a result of different decision-making practices. Our theory suggests, when faced with a health system shock, decision-making can be mapped directly onto three distinct pathways, with each form of decision-making resulting in different consequences for service provision and care. Learning from maternity care delivery throughout the pandemic has demonstrated it is time for care-related, meaningful listening and engagement of staff at all levels to drive forward high-quality care. This can be achieved through capitalising on learning by operationalising thoughts on care and through collective and bold decision-making to innovate in the pursuit of the best quality care possible, whilst avoiding decision-making which will only be tolerated as it disrupts or worse still, devalues care as it is conducted without thought and in reaction to the stressor of a health system shock.

## Supplementary Information

**Additional file 1:** **Appendix 1.** Interview Schedule.

## Data Availability

The datasets generated and/or analysed during the current study are not publicly available due to the sensitive nature of the interviews and the fact that all participants who participated were at one NHS Trust, but could be made available from the corresponding author upon reasonable request.

## Abbreviations

NHS: National Health Service
PPIE: Patient and Public Involvement and Engagement
RCM: Royal College of Midwives
RCOG: Royal College of Obstetricians and Gynaecologists
SARS-CoV-2: Severe Acute Respiratory Syndrome Coronavirus 2 (a.k.a. COVID-19)

## References

[1] Lillie PJ, Samson A, Li A, Adams K, Capstick R, Barlow GD, Easom N, Hamilton E, Moss PJ, Evans A, Ivan M (2020). Novel coronavirus disease (Covid-19): the first two patients in the UK with person to person transmission. J Infect.

[2] UK Government [Internet]. London: Public Health England; c2020–2021. Stay at home: guidance for households with possible or confirmed coronavirus (COVID-19) infection. Available from: https://www.gov.uk/government/publications/covid-19-stay-at-home-guidance/stay-at-home-guidance-for-households-with-possible-coronavirus-covid-19-infection

[3] Coxon K, Fernandez Turienzo C, Kweekel L, Goodarzi B, Brigante L, Simon A, Lanau MM (2020). The impact of the coronavirus (COVID-19) pandemic on maternity care in Europe. Midwifery.

[4] Grünebaum A, Dudenhausen J, McCullough LB, Chervenak FA (2020). Women and children first: the need for ringfencing during the COVID-19 pandemic. J Perinat Med.

[5] Lowe B, De Araujo V, Haughton H, Schweitzer J, Brazil V (2020). Preparing maternity for COVID-19: A translational simulation approach. Aust N Z J Obstet Gynaecol.

[6] Montagnoli C, Zanconato G, Ruggeri S, Cinelli G, Tozzi AE (2021). Restructuring maternal services during the Covid-19 pandemic: early results of a scoping review for non-infected women. Midwifery.

[7] Morau E, Bouvet L, Keita H, Vial F, Bonnet MP, Bonnin M, Le AG, Chassard D, Mercier FJ, Benhamou D (2020). Anaesthesia and intensive care in obstetrics during the COVID-19 pandemic. Anaesth Crit Care Pain Med.

[8] Rocca-Ihenacho L, Alonso C (2020). Where do women birth during a pandemic? Changing perspectives on Safe Motherhood during the COVID-19 pandemic. J Global Health Sci.

[9] Silverio SA, De Backer K, Easter A, von Dadelszen P, Magee LA, Sandall J (2021). Women's experiences of maternity service reconfiguration during the COVID-19 pandemic: A qualitative investigation. Midwifery.

[10] Abdelbadee AY, Abbas AM (2020). Impact of COVID-19 on reproductive health and maternity services in low resource countries. Eur J Contracept Reprod Health Care.

[11] Alsharaydeh I, Rawashdeh H, Saadeh N, Obeidat B, Obeidat N (2020). Challenges and solutions for maternity and gynecology services during the COVID-19 crisis in Jordan. Int J Gynecol Obstet.

[12] Węgrzynowska M, Doroszewska A, Witkiewicz M, Baranowska B (2020). Polish maternity services in times of crisis: in search of quality care for pregnant women and their babies. Health Care Women Int.

[13] De Backer K, Brown JM, Easter A, Khazaezadeh N, Rajasingam D, Sandall J, Magee LA, Silverio SA (2022). Precarity and preparedness during the SARS-CoV-2 pandemic: a qualitative service evaluation of maternity healthcare professionals. Acta Obstet Gynecol Scand.

[14] Jardine J, Relph S, Magee LA, von Dadelszen P, Morris E, Ross-Davie M, Draycott T, Khalil A (2021). Maternity services in the UK during the coronavirus disease 2019 pandemic: a national survey of modifications to standard care. BJOG.

[15] Aziz A, Zork N, Aubey JJ, Baptiste CD, D'alton ME, Emeruwa UN, Fuchs KM, Goffman D, Gyamfi-Bannerman C, Haythe JH, LaSala AP (2020). Telehealth for high-risk pregnancies in the setting of the COVID-19 pandemic. Am J Perinatol.

[16] Fryer K, Delgado A, Foti T, Reid CN, Marshall J (2020). Implementation of obstetric telehealth during COVID-19 and beyond. Matern Child Health J.

[17] Madden N, Emeruwa UN, Friedman AM, Aubey JJ, Aziz A, Baptiste CD, Coletta JM, D'Alton ME, Fuchs KM, Goffman D, Gyamfi-Bannerman C (2020). Telehealth uptake into prenatal care and provider attitudes during the COVID-19 pandemic in New York City: a quantitative and qualitative analysis. Am J Perinatol.

[18] Szabo RA, Wilson AN, Homer CSE, Vasilevski V, Sweet L, Wynter K, Hauck Y, Kuliukas L, Bradfield Z (2021). Covid-19 changes to maternity care: Experiences of Australian doctors. Aust N Z J Obstet Gynaecol.

[19] Greenfield M, Payne-Gifford S, McKenzie G (2021). Between a Rock and a Hard Place: Considering “Freebirth” During Covid-19. Front Global Women's Health.

[20] Jackson L, De Pascalis L, Harrold JA, Fallon V, Silverio SA (2021). Postpartum women's psychological experiences during the COVID-19 pandemic: a modified recurrent cross-sectional thematic analysis. BMC Pregnancy Childbirth.

[21] Jackson L, De Pascalis L, Harrold JA, Fallon V, Silverio SA (2022). Postpartum women's experiences of social and healthcare professional support during the COVID-19 pandemic: a recurrent cross-sectional thematic analysis. Women Birth.

[22] MacGregor R, Hillman S, Bick D (2020). Falling through the cracks: the impact of COVID-19 on postnatal care in primary care. Br J Gen Pract.

[23] Renfrew MJ, Cheyne H, Craig J, Duff E, Dykes F, Hunter B, Lavender T, Page L, Ross-Davie M, Spiby H, Downe S (2020). Sustaining quality midwifery care in a pandemic and beyond. Midwifery.

[24] Romanis EC, Nelson A (2020). Homebirthing in the United Kingdom during COVID-19. Med Law Int.

[25] Montgomery E, De Backer K, Easter A, Magee LA, Sandall J, Silverio SA (2022). Navigating uncertainty alone: a grounded theory analysis of women's psycho-social experiences of pregnancy and childbirth during the COVID-19 pandemic in London. Women Birth.

[26] Silverio SA, Easter A, Storey C, Jurković D, Sandall J, PUDDLES Global Collaboration (2021). Preliminary findings on the experiences of care for parents who suffered perinatal bereavement during the COVID-19 pandemic. BMC Pregnancy Childbirth.

[27] Royal College of Obstetricians and Gynaecologists (UK) (2022). Royal College of Midwives (UK). Coronavirus (COVID-19) infection in pregnancy: information for healthcare professionals.

[28] NHS England (2020). Clinical guide for the temporary reorganisation of intrapartum maternity care during the coronavirus pandemic.

[29] NHS England (2020). Supporting pregnant women using maternity services during the coronavirus pandemic: Actions for NHS providers.

[30] Institute of Health Visiting (UK) (2020). Health visiting during COVID-19: unpacking redeployment decisions and support for health visitors’ wellbeing.

[31] Levers M-JD (2013). Philosophical Paradigms, grounded theory, and perspectives on emergence. SAGE Open.

[32] Annells M (1996). Grounded Theory Method: philosophical perspectives, paradigm of inquiry, and postmodernism. Qual Health Res.

[33] Farrugia B (2019). WASP (write a scientific paper): Sampling in qualitative research. Early Human Dev.

[34] Archibald MM, Ambagtsheer RC, Casey MG, Lawless M (2019). Using zoom videoconferencing for qualitative data collection: perceptions and experiences of researchers and participants. Int J Qual Methods.

[35] Silverio SA, Sheen KS, Bramante A, Knighting K, Koops TU, Montgomery E, November L, Soulsby LK, Stevenson JH, Watkins M, Easter A, Sandall J (2022). Sensitive, challenging, and difficult topics: Experiences and practical considerations for qualitative researchers. Int J Qual Methods.

[36] McIntosh MJ, Morse JM (2015). Situating and constructing diversity in semi-structured interviews. Global Qual Nurs Res.

[37] Silverio SA, Gauntlett W, Wallace H, Brown JM, Clift BC, Gore J, Bekker S, Costas Batlle I, Chudzikowski K, Hatchard J (2019). (Re)discovering grounded theory for cross-disciplinary qualitative health research. Myths, Methods, and Messiness: Insights for Qualitative Research Analysis.

[38] Glaser BG, Strauss AL (1967). Discovery of Grounded Theory: Strategies for Qualitative Research.

[39] Glaser BG (1992). Basics of Grounded Theory analysis.

[40] Strauss AL (1987). Qualitative analysis for social scientists.

[41] Glaser BG (2001). The Grounded Theory Perspective: Conceptualization Contrasted with Description.

[42] Holton JA, Walsh I (2016). Classic Grounded Theory: Applications with Qualitative And Quantitative Data.

[43] Wilson AN, Ravaldi C, Scoullar MJ, Vogel JP, Szabo RA, Fisher JR, Homer CSE (2020). Caring for the carers: Ensuring the provision of quality maternity care during a global pandemic. Women Birth.

[44] Bradfield Z, Hauck Y, Homer CSE, Sweet L, Wilson AN, Szabo RA, Wynter K, Vasilevski V, Kuliukas L. Midwives' experiences of providing maternity care during the COVID-19 pandemic in Australia [published online ahead of print, 2021 Mar 15]. Women Birth. 2021. 10.1016/j.wombi.2021.02.007. 10.1016/j.wombi.2021.02.007 PMC9051255 33752996

[45] Corbett GA, Milne SJ, Mohan S, Reagu S, Farrell T, Lindow SW, Hehir MP, O’Connell MP (2020). Anxiety and depression scores in maternity healthcare workers during the Covid-19 pandemic. Int J Gynecol Obstet.

[46] Melov SJ, Galas N, Swain J, Alahakoon TI, Lee V, Cheung NW, McGee T, Pasupathy D, McNab J (2021). Exploring the COVID-19 pandemic experience of maternity clinicians in a high migrant population and low COVID-19 prevalence country: a qualitative study. Women Birth.

[47] Sheil O, McAuliffe FM (2021). Reorganisation of obstetric services during the COVID pandemic - Experience from National Maternity Hospital Dublin Ireland. Best Pract Res Clin Obstet Gynaecol.

[48] van Manen ELM, Hollander M, Feijen-de Jong E, de Jonge A, Verhoeven C, Gitsels J (2021). Experiences of Dutch maternity care professionals during the first wave of COVID-19 in a community based maternity care system. PLoS One.

[49] Lalor J, Ayers S, CellejaAgius J, Downe S, Gouni O, Hartmann K, Nieuwenhuijze M, Oosterman M, Turner JD, Karlsdottir SI, Horsch A (2021). Balancing restrictions and access to maternity care for women and birthing partners during the COVID-19 pandemic: the psychosocial impact of suboptimal care. BJOG.

[50] Stacey T, Darwin Z, Keely A, Smith A, Farmer D, Heighway K (2021). Experiences of maternity care during the COVID-19 pandemic in the North of England. Brit J Midwifery.

[51] Horsch A, Lalor J, Downe S (2020). Moral and mental health challenges faced by maternity staff during the COVID-19 pandemic. Psychol Trauma Theory Res Pract Policy.

[52] Willan J, King AJ, Jeffery K, Bienz N (2020). Challenges for NHS hospitals during covid-19 epidemic. BMJ.

[53] Ravaldi C, Wilson A, Ricca V, Homer C, Vannacci A (2021). Pregnant women voice their concerns and birth expectations during the COVID-19 pandemic in Italy. Women Birth.

[54] Sanders J, Blaylock R (2021). "Anxious and traumatised": users' experiences of maternity care in the UK during the COVID-19 pandemic. Midwifery.

[55] Sweet L, Wilson AN, Bradfield Z, Hauck Y, Kuliukas L, Homer CSE, Szabo RA, Wynter K, Vasilevski V (2021). Childbearing women's experiences of the maternity care system in Australia during the first wave of the COVID-19 pandemic. Women Birth.

[56] O’Connell M, Crowther S, Ravaldi C, Homer CSE (2020). Midwives in a pandemic: A call for solidarity and compassion. Women and Birth.

[57] Silverio SA, De Backer K, Dasgupta T, Torres O, Easter A, Khazaezadeh N, Rajasingam D, Wolfe I, Sandall J, Magee LA (2022). On race and ethnicity during a global pandemic: An ‘imperfect mosaic’ of maternal and child health services in ethnically-diverse South London. United Kingdom eClinicalMedicine.

[58] Kabesch M, Roth S, Brandstetter S, Häusler S, Juraschko E, Weigl M, Wellmann S, Lang T, Schmidt B, Salzberger B, Ambrosch A (2020). Successful containment of COVID-19 outbreak in a large maternity and perinatal center while continuing clinical service. Pediatr Allergy Immunol.

[59] Chattopadhyay I, Davies G, Adhiyaman V (2020). The contributions of NHS healthcare workers who are shielding or working from home during COVID-19. Future Healthc J.

[60] Bridle L, Walton L, van der Vord T, Adebayo O, Freeman P, Hall S, Finlayson E, Easter A, Silverio SA (2022). Supporting perinatal mental health and wellbeing during COVID-19. Int J Environ Res Public Health.

[61] Fernandez Turienzo C, Newburn M, Agyepong A, Buabeng R, Dignam A, Abe C, Bedward L, Rayment-Jones H, Silverio SA, Easter A, Carson LE, Howard LM, Sandall J, On behalf of the NIHR ARC South London Maternity and Perinatal Mental Health Research and Advisory Teams (2021). Addressing inequities in maternal health among women living in communities of social disadvantage and ethnic diversity. BMC Public Health.

[62] Pilav S, Easter A, Silverio SA, De Backer K, Sundaresh S, Roberts S, Howard LM (2022). Experiences of perinatal mental health care among minority ethnic women during the COVID-19 pandemic in London: a qualitative study. Int J Environ Res Public Health.

[63] Rayment-Jones H, Harris J, Harden A, Khan Z, Sandall J (2019). How do women with social risk factors experience United Kingdom maternity care? A realist synthesis. Birth.

[64] Rayment-Jones H, Harris J, Harden A, Silverio SA, Turienzo CF, Sandall J (2021). Project20: interpreter services for pregnant women with social risk factors in England: what works, for whom, in what circumstances, and how?. Int J Equity Health.

[65] Blumenthal D, Fowler EJ, Abrams M, Collins SR (2020). Covid-19—implications for the health care system. N Engl J Med.

